# Complete chloroplast genome sequence of an endangered plant *Oreocharis cotinifolia* (Gesneriaceae) from Guangxi, China

**DOI:** 10.1080/23802359.2021.1973918

**Published:** 2021-09-15

**Authors:** Jinli Tang, Bo Zhao, Cailin Li, Xin Hong

**Affiliations:** a College of Pharmacy, Guilin Medical University, Guilin, China; b Yunnan Key Laboratory for Integrative Conservation of Plant Species with Extremely Small Populations, Kunming Institute of Botany, Chinese Academy of Sciences, Kunming, China; c School of Leisure and Health, Guilin Tourism University, Guilin, China; d National Gesneriaceae Germplasm Resources Bank of GXIB, Gesneriad Committee of China Wild Plant Conservation Association, Gesneriad Conservation Center of China (GCCC), Guangxi Zhuang Autonomous Region and Chinese Academy of Sciences, Guilin, China; e Anhui Provincial Engineering Laboratory of Wetland Ecosystem Protection and Restoration, School of Resources and Environmental Engineering, Anhui University, Hefei, Anhui, China

**Keywords:** Chloroplast genome Endangered, Oreocharis cotinifolia (Gesneriaceae), Phylogeny

## Abstract

*Oreocharis cotinifolia* is a plant herb with a small population and narrow distribution range in southeast China. It is listed as one of the class 1 key protected wild plants in China and designated as a critically endangered species. In this study, we assembled the complete chloroplast genome of *O. cotinifolia* using data from high-throughput Illumina sequencing. The cp genome is 153,577 bp in length and includes two inverted repeats (IRs) of 25,292 bp, separated by a large single-copy region (LSC) and a small single-copy region (SSC) that are 84,898 bp and 18,095 bp, respectively. The GC content is 37.42%. The genome encodes 128 functional genes, including 87 protein-coding, 37 tRNA, and 4 rRNA genes. Maximum likelihood analysis showed that *O. cotinifolia* is closely related to the congeneric *O. mileensis*. The complete chloroplast genome will contribute to further studies on phylogeny and conservation of *O. cotinifolia* and related taxa in *Oreocharis* of Gesneriaceae.

*Oreocharis cotinifolia* (W. T. Wang) Mich. Möller & A. Weber, previously recognized as a monotypic species *Dayaoshania cotinifolia* W. T. Wang, is an endemic herb native to China (Wang 1983). This species was placed into *Oreocharis* based on a molecular phylogenetic analysis (Möller et al. 2011). Due to its small population size, narrow distribution range and the influence of human activities, its distribution has decreased sharply (Wang et al. 2008; Wang et al. 2013; Wei 2019). It is currently listed as the national class 1 key protected wild plant in the National List of Key Protected Plants (the first batch), promulgated by the State Council in 1999. It was also designated as an extremely endangered species by the Red List of Chinese Species (Wang and Xie 2004). The phylogenetic status of *O. cotinifolia* has been well studied (Weber et al. 2013; Möller et al. 2016), however the complete chloroplast genome of *O. cotinifolia* has not been well studied. Here the complete chloroplast genome was assembled and annotated to contribute to its further systematic study and conservation genetics.

The leaf samples were collected from Dayaoshan Mountain, Guangxi, China (109°54′–110°15′E longitude and 23°43′–24°09′N latitude), and the voucher specimens were deposited in the Herbarium of Guangxi Institute of Botany, Chinese Academy of Sciences (DYS-2019-009, IBK, http://www.gxib.cn/spIBK/, contact person and email: Fang Wen and email is 41617562@qq.com). DNA was extracted following the protocol as described previously (Ling and Zhang 2019). Library construction and sequencing was performed by the Wuhan Bena Biotechnology Co., Ltd. Libraries contained an insertion size of about 400 bp and high throughput DNA sequencing (150 bp on the opposite end) was performed on the Illumina Hiseq 4000 platform to generate the sequence data of about 4 GB. The chloroplast genome sequence of *O. esquirolii* (MT612436) served as the reference sequence, and SPAdes software (version: 3.10.1, parameter: –k 127) was used for genome assembly. Blastn (version: BLAST 2.2.30+, parameter: –evalue 1e–5) was used to confirm the accuracy of the assembly, and the sequences with a retention ratio of more than 1000 bp and a coverage of more than 90% were retained. Joined sequences were annotated using online CPGAVAS2 (http://47.96.249.172:16019/analyzer/annotate), and then inspected manually. The Maximum likelihood tree was inferred using RAXML (version: 8.2.4) with the following parameters: -f a-m GTRCAT-p 12345-x 12345-# 1000.

The length of complete chloroplast genome sequence was 153,577 bp (MN579510), the large single-copy region (LSC) region was 84,898 bp, the small single copy (SSC) region was 18,095 bp, and two inverted regions (IRs) were 25,292 bp for each repeat. A total of 128 genes were predicted, consisting of 87 protein-coding, 37 tRNA and 4 rRNA genes. The total GC content was 37.42%. The The cp genome features of *O. cotinifolia* were similar to other reported species of *Oreocharis* in orientation, order and gene content (Meng et al. 2019; Gu et al. 2020).

In order to ascertain phylogenetic position of *O. cotinifolia* in Gesneriaceae, the chloroplast genome sequences of 24 Gesneriaceae species were downloaded from NCBI GenBank database, and the ML tree was constructed using *Fraxinus sieboldiana and F. insularis* (Scrophulariaceae) as outgroups. Previous phylogenetic studies indicated that *Thamnocharis* embedded into *Oreocharis*, and *T. esquirolii* was a synonym of *O. esquirolii* (Wang et al. 2010; Möller et al. 2011; Meng et al. 2019). Phylogenetic analysis of our study also revealed that *O. cotinifolia* is a sister species to *O. mileensis* (Figure 1), and supported resurrection of the name *O. esquirolii* from *T. esquirolii*. The complete chloroplast genome of *O. cotinifolia* provides data for study of its conservation genetics and the phylogenetic relationship for future studies of the Gesneriaceae.

**Figure 1.**
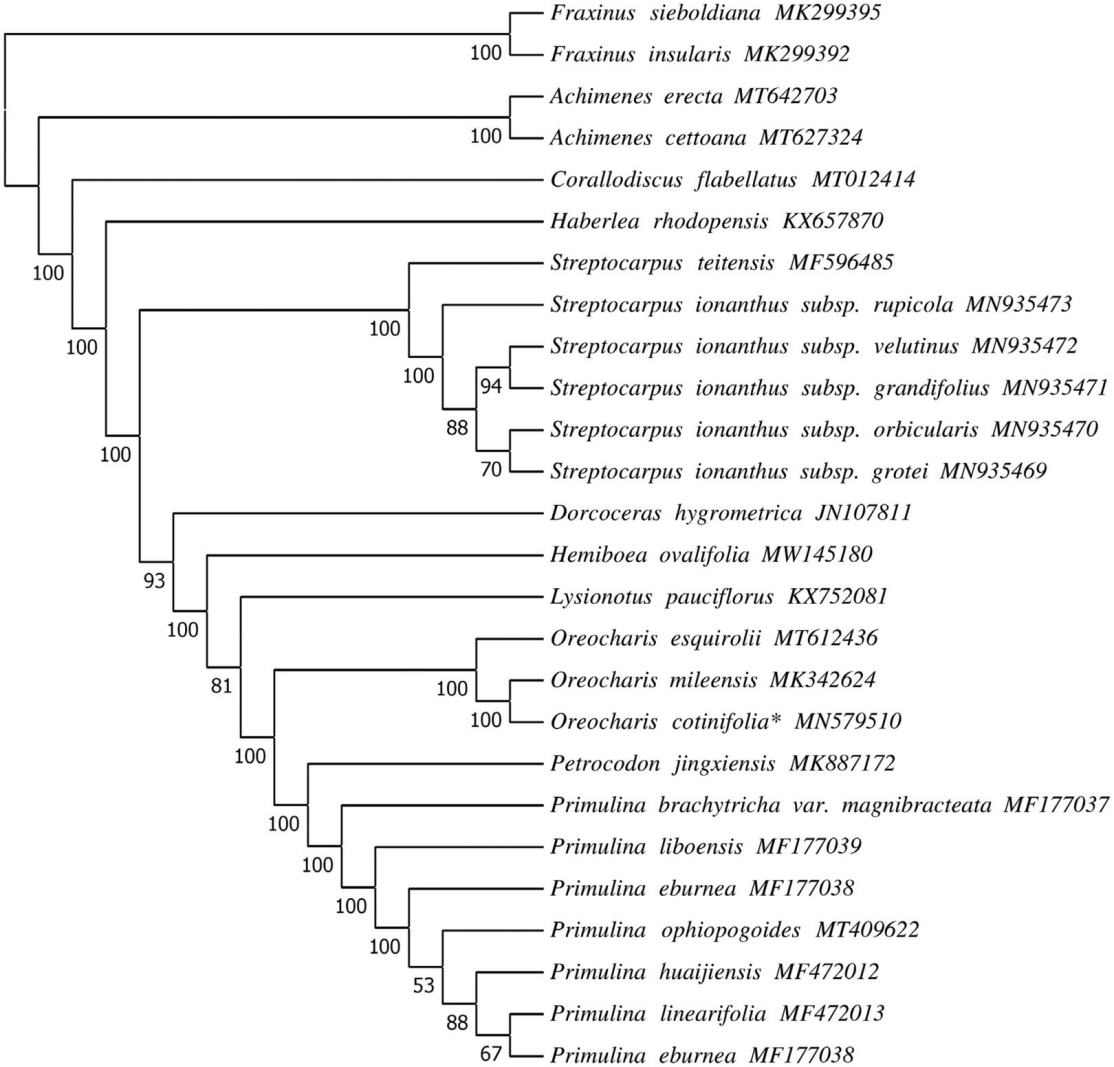
Phylogenetic tree reconstructed by Maximum Likelihood (ML) analysis based on chloroplast genome sequences, including Oreocharis cotinifolia* sequenced in this study. Numbers below branches are assessed by ML bootstrap.

## Data Availability

The raw sequencing data have been deposited in the NCBI Sequence Read Archive under accession numbers PRJNA692635. The data that support the findings of this study are openly available in GenBank of NCBI at https://www.ncbi.nlm.nih.gov, reference number MN579510. The certificate specimens were deposited in the Herbarium of Guangxi Institute of Botany, Chinese Academy of Sciences (DYS-2019-009), contact person is Fang Wen and email is 41617562@qq.com.

## References

[CIT0001] Gu L, Su T, An MT, Hu GX. 2020. The complete chloroplast genome of the vulnerable *oreocharis esquirolii* (Gesneriaceae): structural features, comparative and phylogenetic analysis. Plants. 9(12):1692. 10.3390/plants9121692 PMC7760870 33276435

[CIT0002] Ling LZ, Zhang SD. 2019. The complete chloroplast genome of *Magnolia omeiensis* an endangered and endemic species in China. Mitochondrial DNA Part B. 4(1):1909–1910. 10.1080/23802359.2019.1699878 PMC7748798 33366497

[CIT0003] Meng J, Zhang L, He J. 2019. Complete plastid genome of the endangered species *Paraisometrum mileense* (Gesneriaceae) endemic to china. Mitochondrial DNA Part B. 4(2):3585–3586. 33366096 10.1080/23802359.2019.1677186 PMC7707429

[CIT0004] Möller M, Middleton D, Nishii K, Wei YG, Sontag S, Weber A. 2011. A new delineation for *Oreocharis* incorporating an additional ten genera of Chinese Gesneriaceae. Phytotaxa. 23(1):1–36.

[CIT0006] Möller M, Wei YG, Wen F, Clark JL, Weber A. 2016. You win some you lose some: updated delineations and classification of Gesneriaceae – implications for the family in China. Guihaia. 36(1):44–60.

[CIT0007] Wang HW, Zhang B, Cheng YQ, Ye YZ, Zhang P, Mo NB, Qin KP. 2013. Genetic diversity of the endangered Chinese endemic herb *Dayaoshania cotinifolia* (Gesneriaceae) revealed by simple sequence repeat (SSR) markers. Biochem Syst Ecol. 48:51–57.

[CIT0008] Wang S, Xie Y. 2004. China species red list, Vol 1. Beijing: Higher Education Press.

[CIT0009] Wang WC. 1983. Duo genera nova Gesneriacearum e Sina. Acta Phytotaxonomica Sinica. 21(3):319–324.

[CIT0010] Wang YB, Liang HW, Liang FJ, Qin KP, Mo NB. 2008. The endangered causes and protecting strategies for *Dayaoshania cotinifolia*, an endemic plant in Guangxi. Ecol Environ. 17(5):1956–1960. 2008,

[CIT0011] Wang YZ, Liang RH, Wang BH, Li JM, Qiu ZJ, Li ZY, Weber A. 2010. Origin and phylogenetic relationships of the Old World Gesneriaceae with actinomorphic flowers inferred from ITS and *trn*L-*trn*F sequences. Taxon. 59(4):1044–1052.

[CIT0012] Weber A, Clark JL, Möller M. 2013. A new formal classification of Gesneriaceae. Selbyana. 31(2):68–94.

[CIT0013] Wei YG. 2019. The distribution and conservation status of native plants in Guangxi, China. Beijing: China Forestry Publishing House.

